# The prevalence of food allergy among Aboriginal people in Canada

**DOI:** 10.1186/2045-7022-3-S3-P77

**Published:** 2013-07-25

**Authors:** L Soller, M Knoll, M Ben-Shoshan, DW Harrington, J Fragapane, L Joseph, Y St Pierre, Y St Pierre, S La Vieille, K Wilson, SJ Elliott, AE Clarke

**Affiliations:** 1 McGill University, Montreal, Canada; 2 University of Toronto, Toronto, Canada; 3 Health Canada, Ottawa, Canada; 4 University of Waterloo, Waterloo, Canada

## Background

Data suggest that Aboriginal people may experience lower rates of food allergy compared with the general population. However, there have not been any population-based studies to estimate the prevalence of food allergy among Aboriginal People in Canada. Given this gap in the literature, the goal of this study is to estimate the prevalence of food allergy among Canadian Aboriginal people (First Nations, Métis or Inuit), and to compare these estimates with the general population.

## Methods

We performed a nationwide, cross-sectional telephone survey of all Canadian provinces and territories. Census Canada 2006 data were used to identify postal codes containing a high proportion of Aboriginal people and telephone numbers were randomly selected. The household respondent was queried on whether any household member had a food allergy. Prevalence estimates and 95% Confidence Intervals (CI) were calculated for the nine most common food allergens (peanut, tree nut, fish, shellfish, sesame, milk, egg, wheat, and soy) and for all foods, among individuals reporting Aboriginal status and for the general population.

## Results

Out of 12,747 households contacted, 6,403 responded (50.2% response rate, representing 15,043 individuals), of which 2,264 reported Aboriginal status (15.1% of individuals). All allergies except peanut and fish are more common among the general population than Aboriginal people, although the difference is only significant for tree nut [1.22% (95% CI, 1.00%, 1.44%) vs. 0.57% (95% CI, 0.31%, 0.98%)], shellfish [1.60% (95% CI, 1.35%, 1.86%) vs. 0.93% (95% CI, 0.58%, 1.41%)], milk [1.97% (95% CI, 1.64%, 2.29%) vs. 0.49% (95% CI, 0.24%, 0.87%)], wheat [0.77% (95% CI, 0.57%, 0.96%) vs. 0.13% (95% CI, 0.03%, 0.39%)] and all foods [8.07% (95% CI, 7.47%, 8.67%) vs. 4.90% (95% CI, 4.05%, 5.87%)].

## Conclusion

Our study suggests that the self-reported prevalence of several food allergies is lower in the Aboriginal population as compared with a representative sample of the general Canadian population. The lower prevalence of self-reported food allergy in the Aboriginal population may be attributable to several factors: genetics, differences in dietary habits and the environment, and inequities in access to health care, education and information about food allergy. These findings support a need for better education and access to health care services for Aboriginal communities, and future studies to explore the role of genetics, diet and environment in the development of food allergy.

## Disclosure of interest

None declared.

